# Supplementary material for: Association between oral microbiome and seven types of cancers in East Asian population: a two-sample Mendelian randomization analysis
Source: Front Mol Biosci. 2023 Nov 21;10:1327893. doi: 10.3389/fmolb.2023.1327893 (PMC10702768; doi:10.3389/fmolb.2023.1327893)
Supplement: Supplementary file 1 [file DataSheet1.ZIP › Supplementary Materials/MR plots of saliva/saliva═╝/ovarian cancer/pheno.98_to_ovarian cancer_forest.pdf]

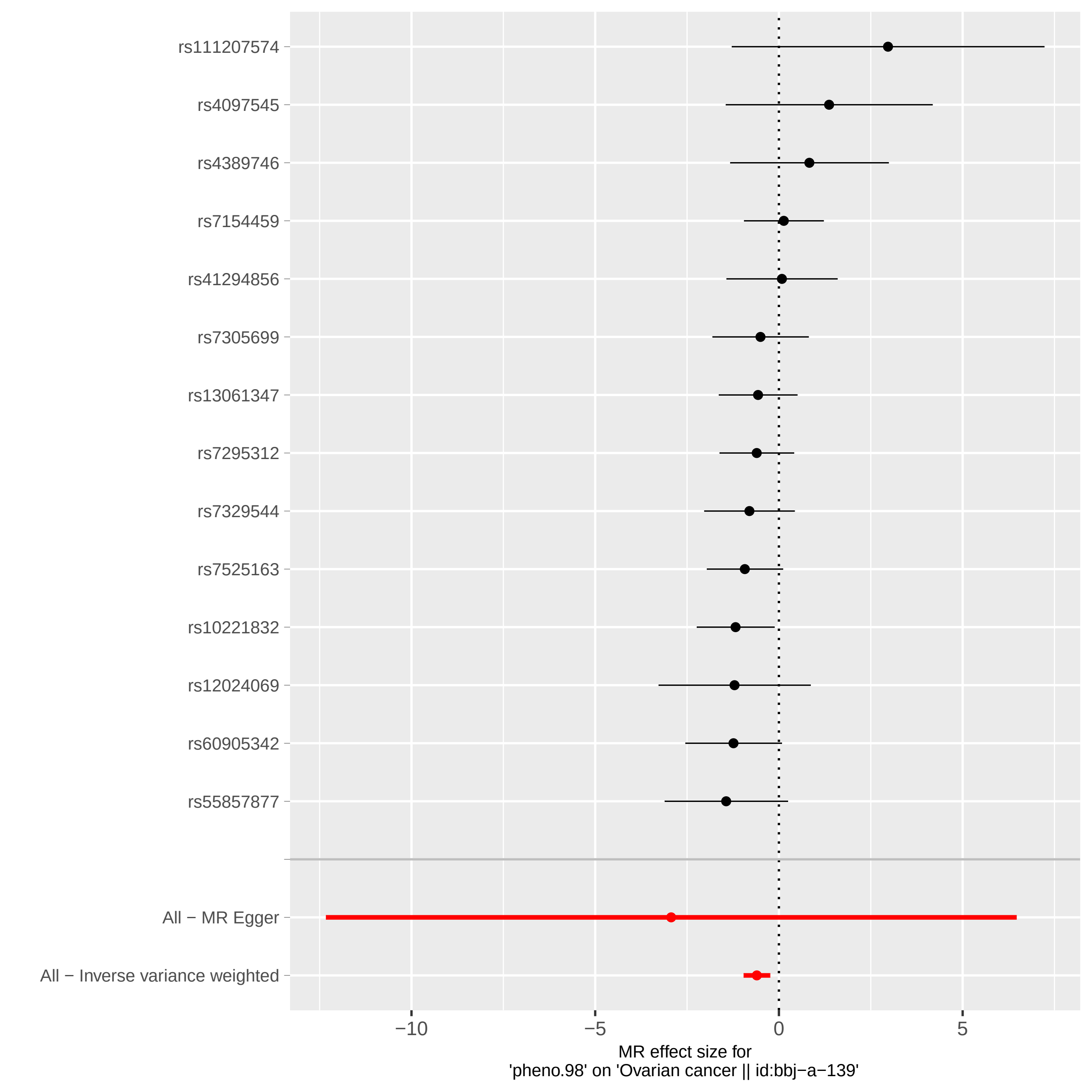
rs111207574
rs4097545
rs4389746
rs7154459
rs41294856
rs7305699
rs13061347
rs7295312
rs7329544
rs7525163
rs10221832
rs12024069
rs60905342
rs55857877
All − MR Egger
All − Inverse variance weighted
−10
−5
0
5
MR effect size for
'pheno.98' on 'Ovarian cancer || id:bbj−a−139'
